# Estimating the extent of glioblastoma invasion: Approximate stationalization of anisotropic advection–diffusion–reaction equations in the context of glioblastoma invasion

**DOI:** 10.1007/s00285-021-01563-9

**Published:** 2021-01-26

**Authors:** Christian Engwer, Michael Wenske

**Affiliations:** Institut für Numerische und Angewandte Mathematik, WWU Münster, Münster, Germany

**Keywords:** Glioblastoma modeling, Stationalization, Reaction–diffusion, 35K57, 92B05, 92C05, 92C50, 92C55

## Abstract

Glioblastoma Multiforme is a malignant brain tumor with poor prognosis. There have been numerous attempts to model the invasion of tumorous glioma cells via partial differential equations in the form of advection–diffusion–reaction equations. The patient-wise parametrization of these models, and their validation via experimental data has been found to be difficult, as time sequence measurements are mostly missing. Also the clinical interest lies in the actual (invisible) tumor extent for a particular MRI/DTI scan and not in a predictive estimate. Therefore we propose a stationalized approach to estimate the extent of glioblastoma (GBM) invasion at the time of a given MRI/DTI scan. The underlying dynamics can be derived from an instationary GBM model, falling into the wide class of advection-diffusion-reaction equations. The stationalization is introduced via an analytic solution of the Fisher-KPP equation, the simplest model in the considered model class. We investigate the applicability in 1D and 2D, in the presence of inhomogeneous diffusion coefficients and on a real 3D DTI-dataset.

## Introduction

Treatment of glioblastoma multiforme (GBM) turmors usually consists of a combination of tumor resection (operation), radio- and chemotherapy (Sathornsumetee et al. 2007). The treatment planning for this type of tumor is particularly challenging, as the medical images do not show a clear boundary and cancerous glia cells infiltrate seemingly healthy tissue far away from the visible center, leading to a diffusive front. Tumor cells have been histologically cultivated from healthy appearing tissue as far as 4 cm away from the bulk of the tumor (Silbergeld and Chicoine 1997). The non invasive medical imaging modalities may only detect the tumor upwards of a finite density threshold of about $16\%$ of tumor volume percentage (Swanson et al. 2008; Patel and Hathout 2017), so that tissues are segmented as healty, although they still contain a significant number of tumor cells.

In clinical practice an average extent of this invisible infiltration of 2 cm normal to the visible tumor is assumed (Chang et al. 2007). One goal of the mathematical modeling of GBM is to estimate the extent of resection and radiotherapy to be applied *outside* of the tumorous regions visible on the medical images.

*Modelling:* Many efforts have been made to mathematically describe the behavior of the tumorous glia cells within the brain. In their review article Alfonso et al. (2017) discuss the evolution of the mathematical modeling of GBM. They present different mathematical approaches which include, but are not limited to monte carlo methods, evolutionary game theory, cellular automata and agent based models. One prominent mathematical approach has been to describe the proliferation and movement of the tumor cells by macroscopic partial differential equations (PDE). These PDE’s are often in the form of diffusion–reaction–advection equations.

The PDE models range from simple reaction-diffusion equations with exponential growth ($\frac{\partial c}{\partial t} = \nabla ({\mathbf {D}}\nabla c) + \rho c$) (Tracqui et al. 1995) to more involved formulations (Jbabdi et al. 2005; Swanson et al. 2011; Engwer et al. 2015, 2016; Hunt and Surulescu 2017; Jan Kelkel 2011; Corbin et al. 2018; Conte et al. 2020).

The PDE forward models strive to describe the full temporal and spatial dynamics of uninterrupted tumor growth. If one wants to use these forward PDE models to predict the tumor invasion in any particular patient, then the parametrization and the initial condition have to be known.

**Fig. 1:**
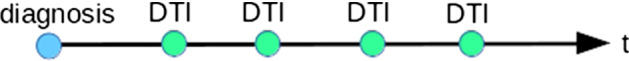
Schematic time line of ideal datasets for the validation of GBM forward models

**Fig. 2:**
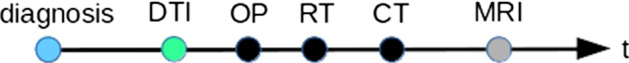
Schematic time line of a realistic sequence of measurements. The initial DTI scan after diagnosis is rapidly followed by a combination of gross tumor resection (OP), radio- and chemotherapy (RT, CT). During treatment, there may be follow-up MRI’s

*Parameters:* For all mathematical models a major challenge is the derivation of model parameters from medical data or experiments. The large number of free parameters in some of the forward models can not be met with experimental data to estimate them with reasonable accuracy. In medical practice the diagnosis of high grade GBM is often rapidly followed by a combination of tumor resection, radio- and chemotherapy, thereby severely altering the growth characteristics of the tumor (Sathornsumetee et al. 2007). In Figs. 1 and 2 we illustrate the discrepancy between an ideal dataset situation for research and the realisitc dataset availability in clinical practice. As an exception to the rule we should mention (Stensjøen et al. 2015) where they were able to measure the growth characteristic (volume increase) of untreated GBM. There have been studies of lower grade gliomas which were left without treatment Mandonnet et al. (2003).

There have been approaches to assess the growth characteristics and parameters in in-vitro experiments, e.g. (Oraiopoulou et al. 2018), but it is an open question whether the information gathered in these experiments is transferable to in-vivo situations. Caragher et al. investigated treatments using novel 3D cell culturing methods in the context of GBM therapy development, but they also remain skeptic of the transferability (Caragher et al. 2019).

Even if in-vitro experiments may prove essential in understanding the involved biochemistry and qualitative effects, their use for the parametrization problem is limited. In order to improve our ability to accurately describe GBM invasion in patients the interlinked problems of parametrization and availability of data have to be addressed. On a patient by patient level, this is a serious challenge.

*Validation:* In order to validate any given forward GBM-model given in the form of advection-diffusion-reaction equations, one would need many time-series of DTI/MR/CT scans of untreated patients. That setup would allow for direct comparison of the in-silico experiments and the medical images of the progressing tumors. One also preferably had these datasets from a large number of representative patients. In order to retrieve such a dataset, one needed to deprive a high number of patients of live prolonging treatment while undergoing regular medical scans. The ethical impossibility is obvious.

The DTI-datasets obtained from a patient with GBM at time of diagnosis are not representative of the healthy state of the subject, as the tumor actively degrades the structure of the brain. Thus guessing initial conditions, i.e., the position of carciogenesis within the degraded DTI-dataset, and running forward simulations can not produce reliable information.

Even in more accessible subjects like rodents, it proved difficult to determine a good parametrization for numerical models. Rutter et al. studied tumor growth in five mice which were injected with tumorous glia cells under controlled conditions. Even with a reportedly careful experimental setup, the resulting tumor sizes varied significantly and fitting parameters of a simple Fisher-KPP tumor model proved difficult (Rutter et al. 2017). Given the goal of improving the ability to accurately describe the tumor invasion in real patients, the problem of validation/falsification has to be addressed. Stationary modeling of the tumor invasion may thus be more appropriate in current situation of missing time series datasets than the full forward modeling.

*Stationary Models:* There have been been approaches to *statically* estimate the tumor’s invasive profile. Notably Konukoglu et al. formulated traveling-time formulations for the tumor invasion problem in the form of eikonal equations that only rely on the imaging data at the time of diagnosis (Konukolu et al. 2006; Konukoglu et al. 2007; E et al. 2010). The results are very promising, as they are perfectly aligned with the imaging information available at diagnosis. It is however not clear how modeling efforts from the forward PDE formulations could be transitioned into the form of eikonal equations.

*Contribution:* We present a stationalization approach, that opens the possibility to estimate the invisible tumor extent at measurement time, building upon existing instationary tumor growth models. By stationalization we refer to the act of deriving approximate stationary formulations for the forward models. The presented method assumes that the tumors growth and diffusive parameters can be estimated from other sources. It may alleviate only part of the parametrization problem by only being sensitive to the relative strength of physical effects and not on their absolute quantitative parametrization. The presented method makes maximum use of the available imaging data. We will first state the class of considered partial differential equations in Sect. 2.1. In Sect. 2.2 we derive a stationalizing term, which allows the reformulation of the forward tumor model into a stationary form. We do this by deriving an analytic expression for the advective term in in the limit of the co-moving 1D Fisher-KPP equation. We present how this stationary formulation, which determines the traveling waves front shape, can be localized to the visible tumor border via the available medical imaging data. We also explain how the analytically derived stationalization term may be used to approximate the tumor density for models that go beyond the Fisher-KPP limit. We numerically verify that the stationalization can be used for any parameter range in one dimension in Sect. 4 via nondimensionalization. We also investigate whether this stationalization approach is viable in the presence inhomogeneous material properties. Finally the advantages and shortcomings of the proposed procedure will be critically discussed in Sect. 5.

## Modeling

### Governing equations

The time-dependent GBM invasion is often modelled by parabolic partial differential equations. We describe the time dependent tumor density with the function $u(x,t) : \varOmega \times T \mapsto \mathbb {R}$, with the d-dimensional domain $\varOmega \subset \mathbb {R}^d$ and a time range $T=[t_0, t_e]$. Here, $t_0,t_e \in \mathbb {R}$ denote the start and the end times. The solution *u*(*x*, *t*) describes the volume percentage of cancerous cells at time *t* and therefore $0\le u(x,t) \le 1$. The dynamic of the density profile is given in the form of a macroscopic partial differential equation. For stationary tumor density distributions we will describe the tumor density with $u_s(x)$.

#### Fully anisotropic advection–diffusion–reaction equation

In this document we focus on two equations. Firstly, the time-dependent fully anisotropic advection–diffusion–reaction equation:

##### Definition 1

Fully anisotropic advection-diffusion-reaction equation

1a $$\begin{aligned} \frac{\partial u(x,t)}{\partial t}&= \nabla ({\mathbf {D}}(x) \nabla u(x,t)) + \nabla ((\nabla \cdot {\mathbf {D}}(x)) u(x,t))&\nonumber \\&+\rho u(x,t)(1-u(x,t))&\text {in~}&\varOmega \times T, \end{aligned}$$

1b $$\begin{aligned} u(x,t_0)&= g(x)&\text {in~}&\varOmega , \end{aligned}$$

1c $$\begin{aligned} \nabla u(x,t)&= 0&\text {on~}&\partial \varOmega \times T. \end{aligned}$$

In (1) the diffusion parameter ${\mathbf {D}}(x)$ is a symmetric positive definite matrix ${\mathbf {D}}(x) \in \mathbb {R}^{d \times d}$ and varies, depending on the spatial coordinate $x \in \mathbb {R}^d$, i.e., the diffusivity can be inhomogeneous and anisotropic. The parameter $\rho \in \mathbb {R}$ is the logistic growth parameter. In order to do any numerical simulations based on (1), a global initial tumor density distribution $g(x)\in \mathbb {R}$ has to be stated, i.e., estimated from medical data. Equation (1 is a prototype model for the invasion of GBM in the sense that many models differ from it merely in the reconstruction of the tumor diffusion matrix ${\mathbf {D}}(x)$ from DTI data, or the addition of other chemotaxis terms (Painter and Hillen 2013). In the following we use (1a) as a prototype tumor model and present how a stationary problem may be formulated whose solution should be very close to that of the forward solution at the time of medical imaging.

#### Limit case: Fisher-KPP equation

In one dimension and for isotropic and homogeneous diffusive properties, Eq. (1) degenerates to the classical Fisher-KPP equation (Kolmogoroff et al. 1988; Fischer 1937).

##### Definition 2

Fisher-KPP equation

2 $$\begin{aligned} \frac{\partial u(x ,t ) }{\partial t } = D\varDelta u(x ,t ) +\rho u(x ,t )(1-u(x ,t )), \end{aligned}$$

again with $x ,t \in \mathbb {R}$ as spatial and temporal coordinates. The diffusion term may be interpreted as the passive diffusive spread of the cells due to a random walk, the rightmost term as a logistic proliferation term, as often encountered in biological contexts. For the initial conditions:

$$\begin{aligned} \lim \limits _{x \rightarrow -\infty }{u(x,t_0) = 1} ,~~\lim \limits _{x \rightarrow \infty }{u(x,t_0) = 0} , \end{aligned}$$

the fisher equation allows travelling wave solutions (Kolmogoroff et al. 1988). These solutions converge over asymptotically long timescales to a wave-front which is constant in shape, moving laterally with a globally constant velocity $v \in \mathbb {R}$. This means, that temporal dynamics are dominated by lateral advection, and not so much by changes of the invasion profiles shape. We will make use of precisely this property.

In higher spatial dimensions, the Fisher-KPP equation has related sigmoid-like traveling wave solutions with circular- or ball-shaped expansion. There are more additional stable wave front patterns in higher dimensions. One of them describes a v-shaped waveform propagating through the two-dimensional medium, which can be interpreted as two straight wave fronts collapsing into each other at a certain angle. Observed in the direction of a bisecting line, this combined wave front is indeed stationary at certain speeds. There also exist spatially oscillating front shapes, but these profiles are only possible for the extension of *u*(*x*, *t*) out of the relevant range $u(x ,t ) \in [0,1]$ (Brazhnik and Tyson 1999). We focus on cases where the wave propagation occurs as a radial expansion from a centered mass.

#### Nondimensionalizations

Since the long term behavior of the 1D fisher equation is that of simple lateral movement, we can state Eq. (2) in equivalent form by introducing new dimensionless variables:

3 $$\begin{aligned}&{\tilde{t}} = \rho t \end{aligned}$$

4 $$\begin{aligned}&{\tilde{v}} = \frac{1}{\sqrt{\rho D}} v \end{aligned}$$

5 $$\begin{aligned}&{\tilde{x}} = \sqrt{\frac{\rho }{D}}x - {\tilde{v}}{\tilde{t}} \end{aligned}$$

The transformation of the spatial coordinate can be understood as a scaled co-moving reference frame, where *v* is the limit propagation speed. Seen from this co-moving reference frame, the wavefront will not move laterally. As time increases, the wave front will converge to it’s limit shape, which we are seeking to reconstruct. After insertion of the new parameters, we find the nondimensionalized co-moving formulation

##### Definition 3

Dimensionless co-moving Fisher-KPP equation

6 $$\begin{aligned} \frac{\partial u({\tilde{x}}, {\tilde{t}})}{ \partial {\tilde{t}}}= \varDelta u({\tilde{x}}, {\tilde{t}}) + u({\tilde{x}}, {\tilde{t}})(1-u({\tilde{x}}, {\tilde{t}})) + v \cdot \nabla u({\tilde{x}}, {\tilde{t}}). \end{aligned}$$

The advective term $v \cdot \nabla u({\tilde{x}}, {\tilde{t}})$ results from the coordinate transformation into the co-moving frame and can be understood as simply counteracting the lateral movement of the propagating front (Ablowitz and Zeppetella 1979; Kolmogoroff et al. 1988). For long times ${\tilde{t}}$ the rate of change $\frac{\partial u({\tilde{x}}, {\tilde{t}})}{ \partial {\tilde{t}}}$ will tend to zero, and the solution of (6) will be effectively stationary. Any laterally shifted limit solution is equally a solution to (6). Therefore, without further side conditions, we do not expect the limit solutions to (6) be unique. The minimum speed $v \in \mathbb {R}$ of the traveling wave solutions has been derived to be $v = 2\sqrt{\rho D}$. Kolomogorov also states that there are solutions for speeds higher than *v*. This fact is due to the reaction term acting independently of the local environment. For the limit of diminishing diffusion, the reaction term will induce very high traveling wave speeds for initial conditions which are close to horizontal. For initial conditions with compact support, which is the relevant case here, the speed tends to $2\sqrt{D \rho }$ as time goes to infinity (Kolmogoroff et al. 1988). In the context of glioma invasion only the very rapid spatial decay of tumor density is relevant and we expect no extended density distributions as initial conditions.

### Stationalization of the Fisher-KPP equation

Based on the comoving formulation of the Fisher-KPP equation and its long term solution, we will derive an approximative problem description in the physical domain. We make use of the fact that in the infinite time limit, we are able to derive the reaction term necessary to calculate the comoving limit solution directly. We then use this reaction term also for stationary problem formulations in the physical domain.

We first derive an alternative form of the advection term in (6), which is exact only in the infinite time limit. We use the limit solution $U({\tilde{x}}): \mathbb {R} \rightarrow \mathbb {R}$ to the co-moving Fisher equation (6). With $U({\tilde{x}})$, we can calculate an analytical expression for $\nabla U({\tilde{x}})$. Also, we may express this gradient in terms of the amplitude of $U({\tilde{x}})$ since the solution is invertible in the relevant range $U({\tilde{x}}) \in (0,1)$. The result is essentially a mapping from the amplitude of $U({\tilde{x}})$ to its gradient. With this analytical expression $p(\cdot )= {\tilde{v}}(\nabla U({\tilde{x}}))(\cdot )$, we may express the problem of finding the limit solution in a stationary way. We use the subscript *s* for solutions $u_s({\tilde{x}}): \varOmega \mapsto \mathbb {R}$ to the stationary problem formulations.

$$\begin{aligned} 0= \varDelta u_s({\tilde{x}}) + u_s({\tilde{x}})(1-u_s({\tilde{x}}))\underbrace{- p(u_s({\tilde{x}}))}_{\text { stationalization}}, \end{aligned}$$

We will now present how this penalty term can be derived from the analytical solution. For ${\tilde{t}}\rightarrow \infty$, no boundary conditions, and the special wave-speed of ${\tilde{v}} = \frac{5}{\sqrt{6}}$, the limit solution is given by

#### Definition 4

Fisher co-moving limit solution

7 $$\begin{aligned} U({\tilde{x}}) = \frac{1}{(1 + \exp (\frac{{\tilde{x}}}{\sqrt{6}}))^2} . \end{aligned}$$

We call that profile the co-moving limit solution (Ablowitz and Zeppetella 1979). The gradient of this limit solution is given by

8 $$\begin{aligned} \nabla U({\tilde{x}})= \frac{ -\sqrt{\frac{2}{3}} \exp (\frac{{\tilde{x}}}{\sqrt{6}}) }{(1 + \exp ( \frac{{\tilde{x}}}{\sqrt{6}} ))^3}. \end{aligned}$$

The inverse of the analytical solution is given by

9 $$\begin{aligned} {\tilde{x}}(U)= \sqrt{6} \ln \Big (\frac{1}{\sqrt{U}} - 1\Big ), \end{aligned}$$

and represents the mapping from a given amplitude $U({\tilde{x}})$ to the corresponding position ${\tilde{x}}$ within the limit solutions profile (7). Substituting the inverse (9) into the gradient expression (8) yields an analytical term for $(\nabla U)({\tilde{x}}(U))$, i.e., a term which is only U-dependent providing the gradient of the wave fronts profile upon evaluation. Thereby we can state a closed form for the stationalization term

$$\begin{aligned} p(U) =|{\tilde{v}}| \sqrt{\frac{2}{3}} (1-\sqrt{U})U. \end{aligned}$$

We have essentially calculated a penalty term, which is only dependent on the local amplitude, and not the local gradient. The penalty parameter $|{\tilde{v}}|$ should be chosen to the same approximate amplitude of the limit speed of the propagation front.

#### Remark 1

The strict equivalence between $p(u({\tilde{x}},{\tilde{t}}))$ and $-|{\tilde{v}}| \nabla u({\tilde{x}},{\tilde{t}})$ only holds in the limit case ${\tilde{t}} \rightarrow \infty$, for which the analytical solution is known and only in the absence of boundary conditions, i.e., $\varOmega \equiv \mathbb {R}$. Although this formulation is only strictly equivalent in case of a completely equilibrated wave form, we will use the derived penalty term as an *approximation* of $|v| \nabla u(x,t)$ at finite times allowing us to define stationalized wave-pinning type models. We present a thorough numerical investigation of the quality this approximation in 4.

#### Definition 5

Stationary Fisher-KPP model

10a $$\begin{aligned} 0&= {\underbrace{\varDelta u_s({\tilde{x}}) + u_s({\tilde{x}}) (1-u_s({\tilde{x}}) }_{\text {tumor model prototype}}}- { \underbrace{ |{\tilde{v}}|~ \sqrt{\frac{2}{3}} (1-\sqrt{u_s({\tilde{x}})})u_s({\tilde{x}})}_{\text {stationalization}}} \text { in } \varOmega , \end{aligned}$$

10b $$\begin{aligned} \nabla u_s({\tilde{x}})&=0 \quad \text { at } \partial \varOmega , \end{aligned}$$

10c $$\begin{aligned} u_s({\tilde{x}})&=0.16 \quad \text { at } \partial \varOmega _i . \end{aligned}$$

Within the encompassing domain $\varOmega$ we define a smaller enclosed domain $\varOmega _{i} \subset \varOmega$ representing the border of the visible tumor on the medical images. At this border we impose an *internal* Dirichlet boundary condition, assuming that the visibility threshold of the tumor is at 16% volumetric tumor density (Swanson et al. 2008; Patel and Hathout 2017). For real datasets this threshold is given by the outer surface of the medical segmentation, for numerical tests we use a solution to the forward problems (2,1a). The additional internal boundary condition on $\partial \varOmega _i$ is used to localize the stationalized solutions.

The Fisher equation is one example out of a family of KPP-type equations which combine a diffusive term with a nonlinear reaction term $f(k): \mathbb {R} \rightarrow \mathbb {R}$. In the case of the Fisher Equation the term is $f(k) = \rho k(1-k)$ with $\rho ,k \in \mathbb {R}$. The reaction term is often chosen in a manner so that it dynamically connects two fixed points of amplitude: $f(0)=0, f(1)= 0, f(k)>0$ for $k\in (0,1)$. Although an exact analytical description of the stable wave fronts proves difficult, the rough characteristic of propagating fronts found in nature (combustion, bacteria growth etc.) is often similar to a sigmoid function. The gradient distribution of any sigmoid-like traveling wave front will have a similar shape as $p(\cdot )$, derived earlier. For any sigmoidal wave-form the gradient amplitude $|\nabla u|$ will be zero for $u=0$ and $u=1$ and of higher amplitude for $0<u<1$.

The underlying idea of the stationalization procedure is, that this gradient distribution, and therefore the penalty term necessary to calculate the traveling wave form stationally, may possibly be approximated to a good degree of accuracy and even for those cases where the analytical solution is not at hand.

### Stationalization of the fully anisotropic advection–diffusion–reaction equation

Returning to the general model in the form of (1), we now define a corresponding stationalized version in dimensional form, using the penalty term derived for the fisher equation in 2.2. The stationalized problem can now be stated as: Find $u_s(x)$ such that

#### Definition 6

Stationary anisotropic advection-diffusion-reaction equation

11a $$\begin{aligned} 0&=\nabla ({\mathbf {D}}(x))\nabla u_s(x))+ \nabla ((\nabla \cdot {\mathbf {D}}(x))) u_s(x) ) \nonumber \\&\quad + \rho u_s(x)(1-u_s(x))- |v|~ \sqrt{\frac{2}{3}} (1-\sqrt{u_s(x)})u_s(x),&\text {in }\varOmega , \end{aligned}$$

11b $$\begin{aligned} \nabla u_s(x)&=0 \quad \text {at } \partial \varOmega ,~ \end{aligned}$$

11c $$\begin{aligned} u_s(x)&=0.16 \quad \text {at } \partial \varOmega _i . \end{aligned}$$

The above problem retains similarity with the general model (1a). It is merely augmented with the penalty term $p(u_s(x))$ and with internal Dirichlet boundary conditions providing the information accessible at imaging time. The matrix ${\mathbf {D}}(x)$ is a reconstruction of the tumor diffusivity from the DTI datasets, $\rho$ is a growth parameter and *v* the penalty parameter. In the limit of homogeneous isotropic diffusion with $\rho =1,D=1$, the above model degenerates to the stationary formulation (10a).

## Numerical methods and error measures

In this section we present the methods used in our numerical experiments to quantify the modeling error of the proposed stationalization. In Sect. 4 we will use the methods to investigate the validity of our approach in a series of problems with growing complexity.

### Numerical scheme

For the time dependent formulations, we follow the method of lines approach to split temporal and spatial operators. For the spatial discretization of both the forward and the stationary formulations we use, for simplicity, a standard first-order finite element discretization on cubic grids with multi-linear trial- and test-functions. We therefore use the same spatial discretization for the comparison between the forward and the stationary formulation. The temporal discretization is an implicit-euler scheme, which is unconditionally stable.The stationalization procedure is largely independent of the chosen numerical discretization. The largest source of error does not lie in the numerical treatment, but in the approximations made in the parametrization, the modeling and in the estimation of the internal Dirichlet constraints. The matrix divergences are pre-calculated by a first order finite difference approximation within each grid cell. The inhomogeneous diffusion matrices at the quadrature points are estimated by taking the value of the nearest neighboring data point.

Positivity of our solution might be violated in finite precision calculations. Let $\omega \in \mathbb {R}$ be a tumor density solution to either the forward or the stationary solutions. Then, whenever the numerical iteration leaves the physically sensible range of $\omega \in [0,1]$, we disregard the reaction term of any given forward- or stationalized model and instead employ the following artificial numerical penalty term

12 $$\begin{aligned} n(\omega )= {\left\{ \begin{array}{ll} -\rho \omega &{}\text {if~} \omega <0\\ \rho (1-\omega ) &{}\text {if~} \omega > 1.\\ \end{array}\right. } \end{aligned}$$

This is done, because a logistic reaction term $f(\omega ) \propto \rho \omega (1-\omega )$ may otherwise amplify numerical fluctuations which produce a slightly negative amplitude.

### Implementation

The implementation was realized within DUNE software framework (Bastian et al. 2008b, a; Blatt et al. 2016). The finite element discretization was implemented within dune-pdelab (Bastian et al. 2010). The non-linear system is solved with a classical Newton–Krylov method, using linear search. The linear systems are solved with an AMG-preconditioned BiCGSTAB solver, using the dune-istl module (van der Vorst 1992; Blatt and Bastian 2007). The release version for all DUNE modules was 2.7.

For the realistic head model the diffusion matrices were reconstructed by the camino software package (Cook et al. 2006).

### Error measure

In Sect. 4.3 we will investigate the impact of the stationalization error on the observed tumor front. In this course we will compare the reconstructed tumor front of a fully instationary simulation with the reconstructed tumor front using the stationalization approach.

**Fig. 3:**
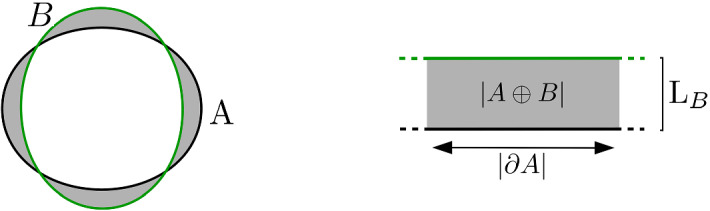
**left:** Symmetric difference region between the two level-sets $|A \oplus B|$: gray regions. **right:** localized sketch of the symmetric difference region, the surface of the level-set volume $|\partial A|$ and the distance between the two level-sets $L_B$

Given a reference density distribution $u_a(x,t): \varOmega \times T \mapsto \mathbb {R}$, and a stationary approximation $u_b(x): \varOmega \mapsto \mathbb {R}$ and a threshold value $\theta \in \mathbb {R}$ we define two domains *A* and *B* as

$$\begin{aligned} A = \{x | U_a(x)\ge \theta \},\qquad B = \{x | U_b(x)\ge \theta \}. \end{aligned}$$

The medically relevant information is the spatial discrepancy between two level-set surfaces ($\partial A, \partial B$) of these density profiles. An absolute measure for this error is the symmetric difference $|A \oplus B|$, as depicted in Fig. 3. It describes those volumes, which are either included *A* but not in *B*, or vice versa. That way, both over- and underestimations of the approximation $u_b$ are represented. The symmetric difference is however not comparable between 1D, 2D and 3D simulations.

The most expressive information in the medical context is the average distance between the two level-sets. We therefore introduce the global characteristic level-set distance.

#### Definition 7

(**Global characteristic level-set distance**) For a given level-set value $\theta$, we define the characteristic level-set distance between $\partial A$ and $\partial B$ as

$$\begin{aligned} L_B := \frac{|A \oplus B|}{|\partial A|}. \end{aligned}$$

It quantifies the average distance between the two level-sets. Assuming a spherical reference geometry for *A* we can approximate this expression in the following way. Given the radius $r_A$, $L_B$ simplifies to

13 $$\begin{aligned} L_B^\text {1d} = \frac{|A\oplus B|}{2}, \quad L_B^\text {2d} = \frac{|A\oplus B|}{2 \pi r_A} \quad \text {and}\quad L_B^\text {3d} = \frac{|A\oplus B|}{4 \pi r_A^2}. \end{aligned}$$

### Artificial imhomogeneous diffusion

We introduce several test cases with randomly perturbed diffusion coefficients. We define the diffusion matrices as

14 $$\begin{aligned} {\mathbf {D}}_\beta = \mathbb {1}_d ~ (\delta )^{\frac{1}{d}}, \end{aligned}$$

with $\delta$ being a uniformly distributed random value: $\delta = \mathrm {unif}(1-\beta ,1+\beta )$. Here, *d* is the spatial dimension. The exponent of $\delta$ is chosen to allow comparisons between the isotropic homogeneous case and the randomized case, by assuring that the average of the determinants of the diffusive medium are close to 1.0 for every realisation of the random field:

15 $$\begin{aligned} |\bar{{\mathbf {D}}}_\beta | = \bar{ (\delta )} \approx 1. \end{aligned}$$

We only compare homogeneous and inhomogeneous cases with the same grid resolution. We evaluate the random inhomogeneous diffusion on the dual grid and assume it to be piecewise constant therein. The diffusivity within one dual grid cell is therefore statistically independent from any neighbor. The effect of statistical scattering of the diffusive properties on the macroscopic front speed is non-trivial. Since the global front speed appears as a linear factor in the analytic derivation of the stationalization term, we may not expect perfect convergence to the analytically derived gradient distribution. The results however give an indication of the effect of realistic datasets.

### Notes on uniqueness

**Fig. 4:**
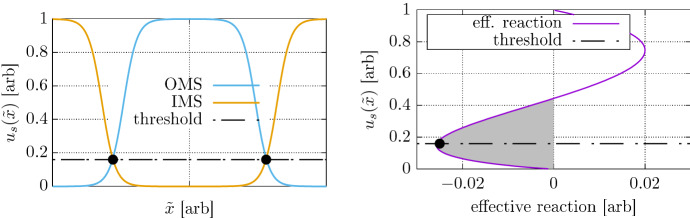
**Left:** Schematic of outward and inward moving solution (OMS, IMS) branches of solutions to 10a, both fulfilling the internal Dirichlet constraints (black dots) at the visibility threshold at $u({\tilde{x}}_c,{\tilde{t}}_e)=0.16$. **Right:** Plot of effective reaction term consisting of the logistic growth and the penalty term. The penalizing regime is indicated in gray. The visibility threshold is within the penalizing regime

The limit solutions to the Fisher Eq. (2) allow for traveling wave solutions moving in both the positive and negative direction. In the stationalized (co-moving) formulation we find a similar situation. Since we will later use an internal Dirichlet condition in the form of $u({\tilde{x}}_c,{\tilde{t}}_e)=0.16$ with ${\tilde{x}}_c\in \partial \varOmega _i$, we may also expect two possible solutions to the comoving formulation (10a). The first stationary solution corresponds to the traveling wave which moves outwards from the tumor center, at that point in time where it satisfies the Dirichlet constraint and is the relevant one in the given setting. We call this the outwards moving solution branch (OMS). The second possible solution corresponds to a traveling wave moving into the constrained region, i.e., the inwards moving solution (IMS). This solution branch is irrelevant to tumor growth modeling. Two stationary solutions of the two branches are sketched in Fig. 4.

When considering the effective reaction term consisting of the logistic growth combined with the penalty term, we find that it has a penalizing regime for $0<u_s({\tilde{x}})<\frac{4}{9}$ and a growth regime for $\frac{4}{9}<u_s({\tilde{x}})< 1$ (Fig. 4). The diffusive process transports mass from high amplitudes to lower amplitudes. The combined reaction term counteracts this process. The visibility threshold, and therefore the constraints, lie within the penalizing regime. In order to select the relevant OMS solution branch in the stationary formulation, we use initial guesses $u_s({\tilde{x}}) \ll 1$ which are well within the purely penalizing regime, so that the newton iteration converges to the outward moving solution reliably.

## Numerical results

We present the results of the numerical validation studies of the stationalization procedure in 1D and 2D. We use the nondimensional formulations only in the homogeneous case, and explicitly state the models we used and the parameters chosen for the test cases with inhomogeneous diffusion. We first compare 1D forward simulations of the homogeneous Fisher-KPP equation with their stationary counterparts and explain the intended procedeure and the numerical setup. After that we compare the gradient distributions in 1D and 2D of forward simulations with the analytical expression derived in Sect. 2. We present an easily reproducible 2D example in 4.3.1. Finally we present the applicability via simulations with a real 3D DTI dataset in 4.4.

### 1D front reconstruction

We first show results for a 1D case. We compare two forward solutions of the models defined in Eqs. (2) and (1) with the solutions of their respective stationalized problem formulations (10a) and (11a). In the nondimensionalized case, we chose the penalty parameter ${\tilde{v}}$, which is the limit speed of from the analytical solution (7). A penalty parameter higher or lower than that would result in steeper or flatter reconstructed profiles. We use the end times ${\tilde{t}}_e,t_e =20$. In the homogeneous case we compare both the stationalized, and the forward simulations in nondimensionalized form (10a). This implies that the stationalization method can be used for all sensible magnitudes and combinations of $\rho$ and *D*. We start the simulations from a small centered gaussian initial condition, and at $t_e$ compare the two solution profiles against each other. The internal boundary condition within the stationary problem (10c) is set at those points, where the forward solution reaches amplitudes higher than 0.16 at $t_e$, i.e., at those points where the tumor border would be visible on an imaging machine with that threshold. Similarly, we show the comparison of a solution to the forward model incorporating inhomogeneous diffusion coefficients (1a) with the now inhomogeneous stationalized formulation (11a). By introducing inhomogeneous diffusive coefficients, the myopic drift term will produce small contributions to the equation.

**Fig. 5:**
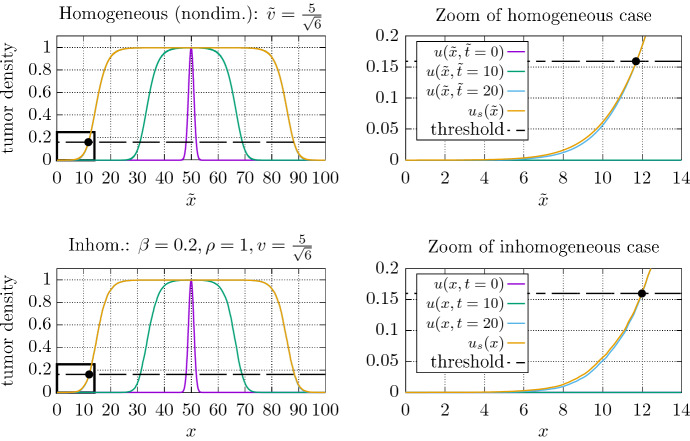
Direct comparison of 1D forward simulations of the nondimensionalized Fisher-KPP equation with its stationalization (10a) and an example with inhomogeneous coefficients (1) and its stationalization (11a). The black dots indicate the internal Dirichlet constraints (10c,11c) given by the simulated imaging threshold ($16\%$) on the forward simulations. The domain was discretized into 1000 equidistant elements. **Top left.:** Initial condition, and states of the (nondimensionalized, homogeneous) forward simulation. **Top right.:** Zoom of the forward solution and the corresponding stationalization. **Bottom left.:** Initial condition, and states of the forward simulation (inhomogeneous). **Bottom right.:** Zoom of the forward solution and the corresponding stationalization (inhomogeneous). Both the forward and the stationalized solutions show slight deviations from a smooth decay in the inhomogeneous case

Figure 5 shows the direct comparison of forward simulations with their stationalized counterparts. This numerical setup shall illustrate how the stationalization can be used to extrapolate the tumor density outside of the known region. Here, the forward simulations represent a fictional gold standard, of which we assume knowledge only above the visibility threshold. By knowledge of these locations, we may provide pinning information in the form of internal Dirichlet conditions to the stationalized formulations and solve for the most likely density distribution on the outside.

In the homogeneous case, the snapshots at different times of the forward solution suggest, that the rough form of the advancing front is formed rapidly. If the forward solution converges rapidly towards the form of the analytical solution, with only diminishingly small corrections to the wave fronts shape at larger times, then correspondingly the approximation of the gradient will perform well even early in the simulation. The direct comparisons in Fig. 5 show that the stationalization produces a reasonable approximation to the full forward solutions. The inhomogeneous coefficients induce small deviations from a smooth front shape, which are present in both the forward simulations profile as well as in the stationalized solution. It is to be expected that wherever the internal constraint results from thresholding of an underlying smooth distribution, the stationalization will perform well since the real density distribution is close to the equilibrated wave-form. The one dimensional setup is not very realistic, but practical to illustrate the procedure.

### Investigation of modeling error

We will numerically investigate the effect of inhomogeneous diffusion on the gradient distribution, and by this the applicability of the chosen stationalization term. As the analytic formulation of $(\nabla U)(U)$ is only valid in the homgeneous 1D limit case, we expect a modeling error, which we will assess in different test cases.

#### 1D gradient distributions

We consider a one dimensional forward simulation of Eq. (1) starting from a Gaussian initial condition in the center of a one dimensional domain $x' \in [0,200]$. We first simulate the homogeneous case with $D=1,\rho =1$ where we expect perfect convergence of the gradient distribution to the analytical expression $(\nabla u(x,t))(u(x,t)) = p(u(x,t))/|v|$ as derived in (10). In the homogeneous case there are no advective terms active, as $\nabla \cdot D(x)$ is zero. Upon start of the simulation, two traveling waves form and move away from the center of the domain. After a short initial phase, the wave-fronts in the homogeneous medium asymptotically approach the front shape of the analytical solution (7) and its symmetric counterpart. The fronts speed and its shape within the inhomogeneous material are slightly perturbed. Similarly the relation $(\nabla u) (u)$ is disturbed.

**Fig. 6:**
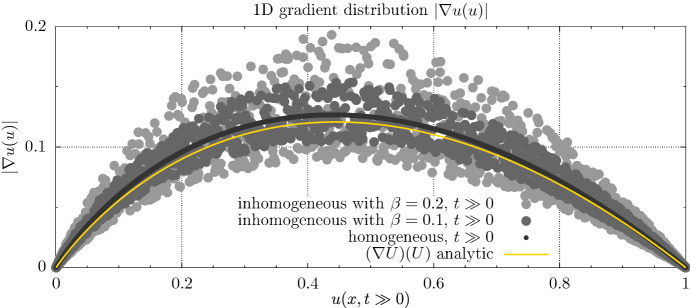
Scatter plot of 1D gradient distributions: magnitude of gradient of 1D wave-front profile. The gradient profile of the homogeneous numerical solution approaches the analytic expression only at asymptotically large time-scales. The underlying characteristic is not destroyed by the introduced inhomogeneities

In Fig. 6 we investigate how the gradient distributions approach the analytical expression for the homogeneous and inhomogeneous case and how strongly it differs from the analytical expression in the case of heterogeneous coefficients.

#### 2D gradient distributions

We consider a square domain ($L_x = L_y=200$) in 2D with a Gaussian initial condition in the center. We again simulate a forward solution to (1). After the start of the simulation, the wave-front circularly propagates outward from the central point. Slow convergence of the gradient distribution to the analytic expression (10) can be replicated in the 2D homogeneous case. In the inhomogeneous case we observe analog deviations from the predicted distribution.

**Fig. 7:**
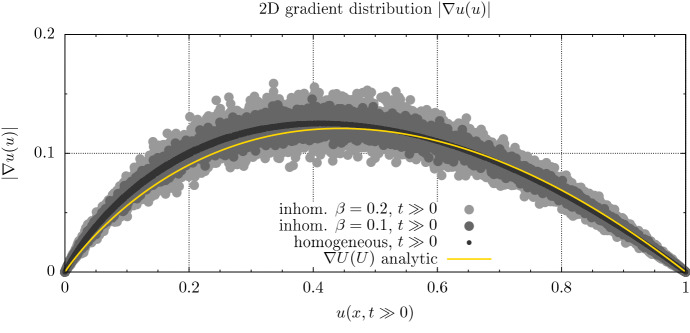
Gradient distribution of a 2D simulation with different inhomogeneous diffusive properties ($\beta = 0.0, 0.1, 0.8$), see (14)

Contrary to the one dimensional case, there is an effect of curvature in higher dimensions slowing the convergence towards the 1D gradient distribution. However in the limit case the wave propagation still reduces to a 1D dynamic in the propagation direction (JDMurray 2007). It is obvious that the introduction of random material properties breaks the strict applicability of the stationalization term. However Fig. 7 suggest that also in 2D the underlying polynomial relation between the wave-fronts amplitude and its gradient is merely perturbed by the material properties. Compared to the usual parameter uncertainties, we consider this modeling error small and thus a stationalized solution, making use of the analytical gradient distribution, should still provide reasonable estimates on the density profile. Since the global propagation speed *v* appears as a linear factor, any process that alters the propagation speed away from the analytical value should have a linear effect on the gradient distribution.

### Effect on estimation of the tumor front

The stationalization includes a modeling error due to the imperfect approximation of $\nabla u$. The numerical observation in Sect. 4.2 suggests that the average behavior is still well described by our analytic reformulation 10a. In the following we will investigate the impact of this stationalization error on the actual tumor front. We will use test cases with growing complexity.

We try to mimic the situation observed in the medical application and present the procedure of estimating the tumor extent at the time of diagnosis. To generate artificial datasets in controlled scenarios, we simulate the carciogenesis by first assuming a small Gaussian initial condition. Secondly, we propagate the density profile for a certain time simulating the uninterrupted tumor growth. Finally, at the time of diagnosis, we use a level-set on the forward solution of $16\%$ to represent the thresholded medical imaging modalities. Other choices of threshold value are possible. We then use the thresholded volume as an internal Dirichlet constraint and solve the stationary problem (11a). In this numerical setup both the solution to the forward problem *u*(*x*, *t*) and the solution to the stationalized problem (11a), $u_s(x)$ are calculated and compared. In any real world situation only the thresholded image information would be accessible.

#### 2D butterfly test case

In order to assess the viability of the stationalization for more realistic tumor models we now move to two dimensions with inhomogeneous coefficients (Eq. (11a)). We set up an inhomogeneous but isotropic field for the diffusion matrix by scaling the unit matrix according to its $x'_1$ position

16 $$\begin{aligned} {\mathbf {D}}(x') = \mathbb {1}_2~(1.0+\sin \Big (\frac{3 \pi }{L_x} x'_1\Big )~0.9). \end{aligned}$$

This effectively separates the domain in three distinct regions, with the left- and rightmost third of the domain having higher diffusivity and the middle strip having reduced diffusivity. The changes in diffusivity may represent gray and white matter regions in a primitive way. In this example there is more long-range deviation of the diffusive properties than in the 1D examples in Sect. 4.1. We again chose the penalty parameter $v= \frac{5}{\sqrt{6}}$ and $\rho = 1$. In a medical situation the task is to estimate the region and intensity of radiotherapy to be applied and the area of resection from only the thresholded information visible at the time of diagnosis.

**Fig. 8:**
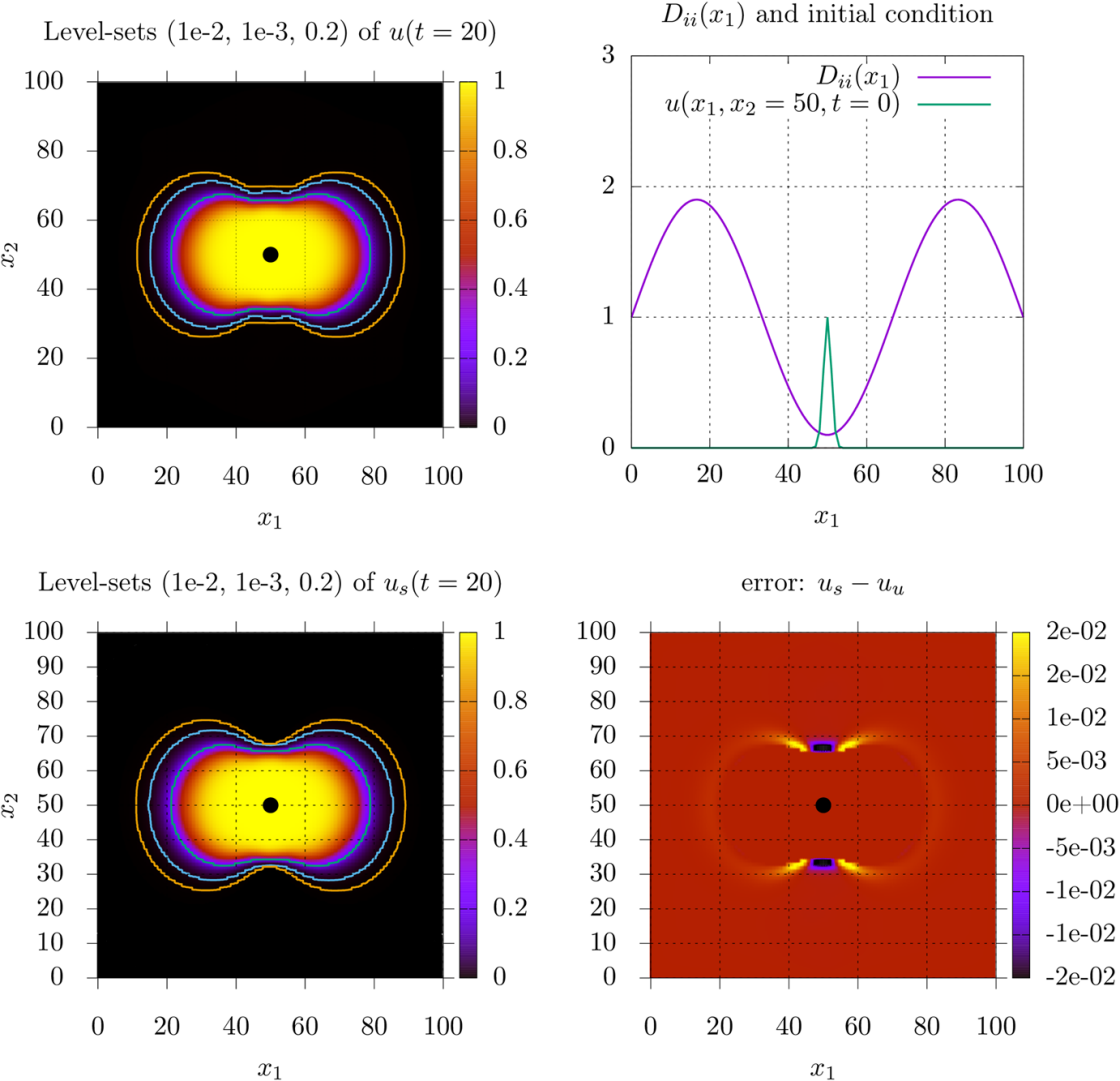
Numerical simulation of (1a) in an inhomogeneous isotropic test case. **Top left:** level-sets (white) (outside to inside: 1e-3,1e-2,0.16) on the forward solution at t=20. The innermost level-set at u=0.16 serves as the internal Dirichlet constraint (11c) for the stationary simulation. **Top right:** amplitude of the identical diagonal entries $D_{ii}$ of the diffusivity matrix as given in (16) and horizontal cut through the Gaussian initial condition at $x=(50,50)$. **Bottom left:** level-sets (white) (outside to inside: 1e-3,1e-2,0.16) on the stationary solution, the solution is constrained beginning from the level-set u=0.16. **Bottom right:** Error field indicating regions and the amplitudes of over- and underestimation

Figure 8 compares predictions of the forward simulation of Eq. (1) with the stationary solutions of Eq. (11a). The stationalization greatly benefits from the localization provided by internally constrained region. Since most of the tumor mass is above the visibility threshold, the stationalization only has to provide the estimation on the surrounding region. Similar to the 1D constrained situation, the stationalization captures the profile quite well, but slightly overestimates the invasion extent in low density regions.

### 3D stationalization for a realistic dataset

To show the applicability on real patient data, we use the publicly accessible DTI-dataset provided by the camino^1^ software project (Cook et al. 2006). We relate the tumor diffusion to the water diffusion by a simple scalar factor:

17 $$\begin{aligned} {\mathbf {D}}(x) = \alpha {\mathbf {D}}_w(x). \end{aligned}$$

**Fig. 9:**
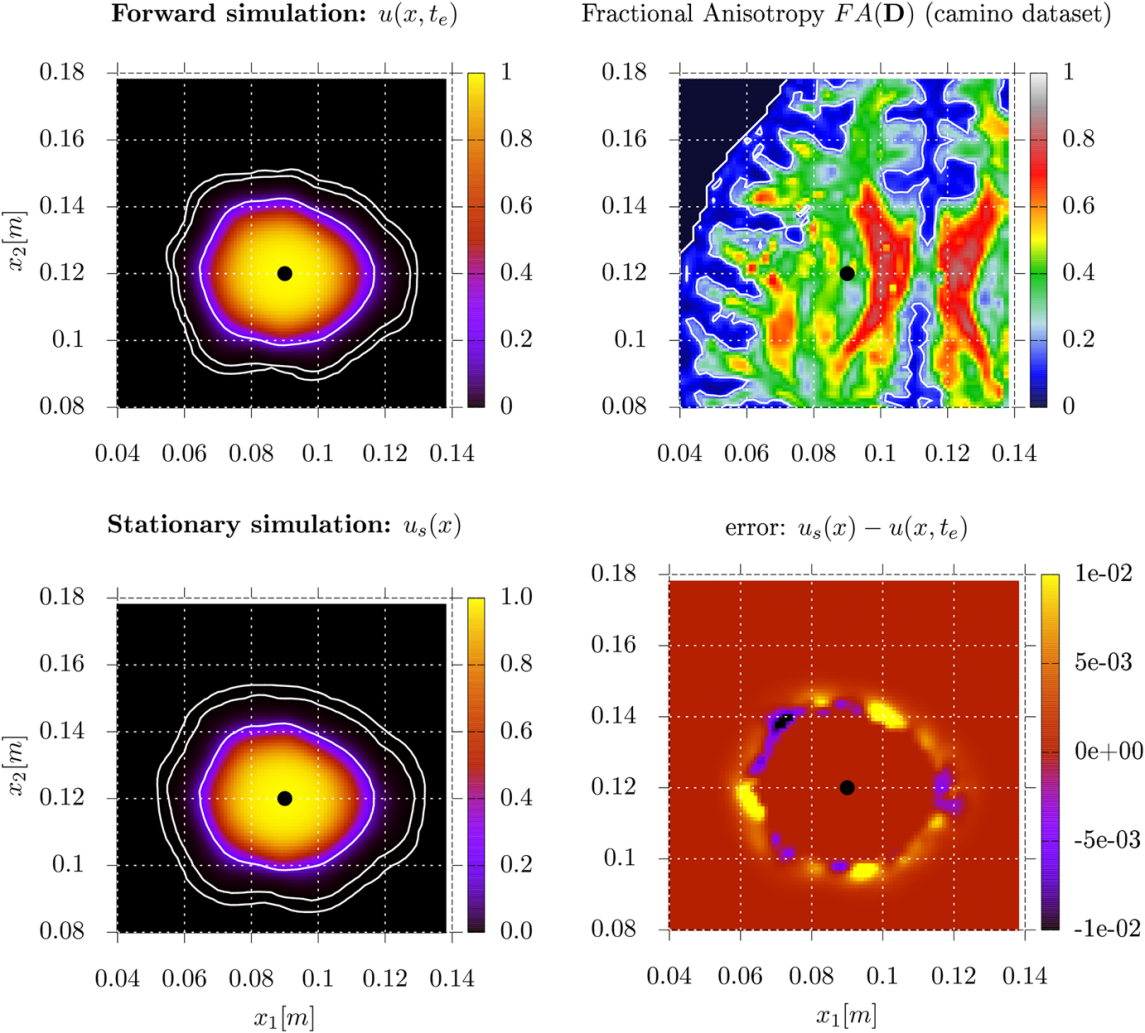
Horizontal slice of the 3D results and dataset. The black dot indicates the position of the small Gaussian initial condition at $x= (0.09m,0.12m,0.05m)$. **Top left:** level-sets (white) (outside to inside: 1e-4,1e-3,0.16) on the forward solution at 90 days. The innermost level-set at $u(x,t_e)=0.16$ serves as the internal Dirichlet constraint (11c) for the stationary simulation. **Top right:** Fractional Anisotropy of the reconstructed tumor diffusion matrix ${\mathbf {D}}(x)$ from the camino dataset (Cook et al. 2006). **Bottom left:** Identical level-sets on $u_s(x)$. **Bottom right:** Regions of over- and underestimation by the stationary solution

**Fig. 10:**
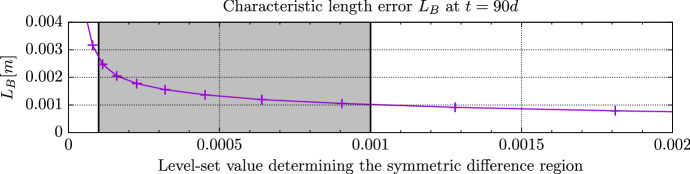
Characteristic length errors $L_B$ for given level-set values for the 3D example camino dataset , see (13). The gray region indicates the errors between the level-sets used in Figure 9

More advanced reconstructions are possible, but not central to this example. A variety of different tumor diffusion models has been proposed in the literature, see e.g. (Hunt 2018; Painter and Hillen 2013; Conte et al. 2020). We use the forward model (1) and the corresponding stationary problem (11a), with the parameters in Table 1 to scale the equations to a realistic range. Here $\alpha$ is a dimensionless parameter, *v* the penalty parameter, and $\rho$ is the growthrate. We again follow the procedure described in Sect. 4.3 and start a forward simulation from a small Gaussian at $t_0 = 0$ until $t_e = 90d$, and use a level-set ($u(x,t_e)=0.16$) as the constrained region for the stationary formulation. Figure 9 shows the direct comparison of the forward simulation and the stationalization. All local extentions or reductions induced by the local increase or decrease of the underlying diffusivity are present in both the forward and the stationalized solution. For this particular example, we measured the characteristic level-set distance $L_B$ as given in (13) for a series of small level-set values. Figure 10 indicates that the average distance error is between $1-2.5\text {mm}$ for the level-sets chosen in our numerical example. These level-sets were chosen to replicate the current treatment radius of about 2cm Chang et al. (2007).

**Table 1:** Parameters used to scale the terms in (1) and (11a) to realistic ranges

**Parameter**	**Value**
$\alpha$	5e-12
$\rho$	1e-6 [1/s]
*v*	2.04e-6 [m/s]

## Discussion

We presented a stationalization approach for the estimation of the glioblastoma invasion extent. The stationalization approach partially addresses the problems of parametrization and data availability. The stationary simulations do not depend on the *complete* knowledge of the initial condition to produce reasonable tumor invasion estimates. The thresholded information provided by the medical images, i.e., the medical segmentation, might be fully utilized with the limited information it provides. Typically the segmentation provides a classification of tissue into a number of categories, i.e., gray matter, white matter, edema, necrotic etc. The segmented border between tissue categorized as healthy, and that which is categorized as tumorous can be directly used to provide the internal Dirichlet constraints as in 11c. The stationalization only requires datasets from one point in time, i.e., one DTI scan and a medical segmentation of the tumorous region. This is the data that is routinely gathered in medical practice, as it is used for planning the radiation therapy and resection. Stationary simulations, as presented here, may provide an additional tool in this regard without altering the imaging practices.

The problem of quantifying model parameters is also partly alleviated by the fact that in the stationary formulation the solution depends merely on the strength of the parameters with respect to each other, and not their absolute values. It may be easier to experimentally determine ratio values between the three important parameterized terms: ${\mathbf {D}}, v$ and $\rho$ than the full set of parameters needed for a forward simulation. Forward models which fit into the form of Eq. (1) may only produce reliable results if the correct initial condition *g*(*x*) is known and the parameters are determined to a reasonable degree of accuracy. In the medical setting the only information at hand is the medical imaging at time of diagnosis. For the forward models to produce reasonable estimates on the tumor invasion profile we would firstly need information on the location and time of the carciogenesis, secondly the information on the non-degraded diffusive properties of the tissue surrounding it and lastly the correct parametrization on a per-patient basis. We want to emphasize that the temporal dynamics of the tumor growth, although scientifically interesting, are not necessarily relevant in the medical treatment planning. The information needed for treatment with the current techniques is an accurate description of the tumor density field *at time of diagnosis*.

The derivation of the stationalization term for the one dimensional Fisher equation is not strictly transferable to tumor models which incorporate medical data or have additional advection terms, but the results from Sect. 4.4 indicate that they may still produce reasonable predictions. If the underlying diffusion matrix field included strong inhomogeneities inducing strong advective terms, the procedure might lose its validity, however an investigation of the given 3D DTI dataset shows that the Péclet number relating the drift and diffusion strength as given in (11a), $\tau = \frac{|b~L|}{|D|}$, stays mostly below 0.3 when ${\mathbf {D}}$ is derived from the DTI data by simple scaling (17). Here, *b* is the local advective magnitude, *L* is the cell spacing and |*D*| is the Frobenius norm of the local diffusivity. Most tumor models show traveling wave characteristics, where the main physical effects include diffusion and nonlinear growth. We recommend close inspection of both the gradient distributions and the Péclet numbers if the stationalized model should be extended. The stationalization procedure should retain its applicability as long as the gradient distribution retains its underlying characteristic as visible in Fig. (7), and the distance between medical segmentation and the border induced by the level-sets is not chosen too large. In the case that the presented approximation fails, it may also be possible to introduce more elaborate numerical ways to fit the necessary penalty term locally.

We compared the level-sets of the forward simulation with those of the stationary simulation and found characteristic distances between them of about $L_B \approx 1.0-2.5$ mm (Fig. 10). Compared to a fixed-size radius of 2cm around the visible tumor (Chang et al. 2007), these errors seem justifiable. It should, of course be possible to find an optimal penalty parameter *v* for a given set of forward model parameters. A medical practitioner might choose the actual level-set value to replicate the current practice of treating a 2 cm radius around the bulk tumor and then use qualitative information in the form of locally recommending an extension or retraction of the treatment radius. The stationary model will correctly capture the effect of the material properties, as presented in Fig. 8. Where the tissue is more diffusive, the level-set on $u_s(x)$ will overextend the 2 cm radius and where the diffusivity is small, more brain tissue might be left untreated. If the underlying tumor model should be extended, all effects increasing- or diminishing the spread of glia cells will be reflected accordingly in the stationalized solution.

Should time-series datasets become available, then the stationalization may be used to estimate the initial condition *g*(*x*) for a forward simulation from the first dataset. An initial condition calculated in this way should be closer to a presumably smooth real density profile than using a stepped profile with steep gradients.

Naturally the computation of solutions to the stationary problem take less time than a full forward simulation. While compute servers are usually available in academic institutions, the possibility to calculate the results on a regular consumer pc with only short computation times is important in order to transfer such methods into clinical application. The stationary simulation allows for the computation of sets of solutions for varying parameters within a short time frame. We present exemplary run times for the camino dataset on recent hardware for the two cases in Table 2.

**Table 2:** Runtimes for the test cases in two- and three dimensions on different hardware

**Simulation type**	**Hardware**	**Run time**
2D, 90 days	Intel i5-7200U ( 4x2.50GHz )	42 s
2D, stationary	Intel i5-7200U ( 4x2.50GHz )	0.4 s
3D, 90 days	Intel i5-7200U ( 4x2.50GHz )	1:31 h
3D, stationary	Intel i5-7200U ( 4x2.50GHz )	76 s
3D, 90 days	AMD EPYC 7501 (32x 2.0GHz)	18 min
3D, stationary	AMD EPYC 7501 (32x 2.0GHz)	9 s

### Outlook

There might be further improvements to the stationalisaton approach. Altering the stationalization term, which currently assumes a globally constant wave-propagation speed, to be sensitive to the local material properties might further improve the results. The proportionality of the wave speed ($v\ge 2~\sqrt{D \rho }$) in the one dimensional Fisher-KPP equation may be an indicator for how a localized penalty parameter could be improved. Instead of choosing a constant *v* globally, it might be possible to set a penalty factor linearly combined with local information. Thereby incorporating the local increases and decreases in diffusivity into the stationalization.

The formulation presented here is based on the solution stated in Ablowitz and Zeppetella (1979). It might also be possible to find analytical expressions for approximate solutions like the perturbation solution stated in JDMurray (2007). Including only leading order does not lead to reasonable penalty terms, but inclusion of higher orders might be possible.

In Sect. 4.4 we used a real dataset, but a comparatively primitive tumor model. It should be possible to extend the stationalization procedure to incorporate additional effects like chemo- or haptotaxis as long as the dynamic of producing traveling wave solutions of sigmoidal shape is not altered by the additions. In the example in Sect. 4.4, we used a level-set on the forward simulation as the internal Dirichlet constraints for the stationalization. In a medical setting one would directly use the medical segmentation from the DTI/MRI modalities. There are promising advances in generating tumor segmentations in an automated fashion, e.g. BraTumIA^2^ (Porz et al. 2014).

Automating the process of the segmentation opens up the possibility to use a fully automated process to advise the treatment planning in real patients.

We showed how well a stationalized formulation would perform compared to a forward simulation if all the necessary information were present. The fact that time-series datasets are largely unavailable and therefore no parameterizations can be derived from them, makes direct comparisons between existing tumor models difficult. It is, however possible to compare the stationalized versions of existing models with only the datasets from the time of diagnosis. One could then compare these level-sets to the clinical target volume (CTV) regularly produced in medical practice. This offers an attractive approach to perform a model comparison for a wide range of tumor models proposed in the literature.

## Footnotes

1 http://camino.cs.ucl.ac.uk/index.php?n=Tutorials.DTI.

2 https://www.nitrc.org/projects/bratumia.

## References

[CR1] Ablowitz MJ, Zeppetella A (1979). Explicit solutions of fisher’s equation for a special wave speed. Bull Math Biol.

[CR2] Alfonso J, K T, M S, B K, A HD, KR S, H H, A D (2017) The biology and mathematical modelling of glioma invasion: a review. J R Soc Interface 10.1098/rsif.2017.0490 PMC5721156 29118112

[CR3] Bastian P, Blatt M, Dedner A, Engwer C, Klöfkorn R, Kornhuber R, Ohlberger M, Sander O (2008). A generic grid interface for parallel and adaptive scientific computing. Part II: implementation and tests in DUNE. Computing.

[CR4] Bastian P, Blatt M, Dedner A, Engwer C, Klöfkorn R, Ohlberger M, Sander O (2008). A generic grid interface for parallel and adaptive scientific computing. part I: abstract framework. Computing.

[CR5] Bastian P, Heimann F, Marnach S (2010). Generic implementation of finite element methods in the Distributed and Unified Numerics Environment (DUNE). Kybernetika.

[CR6] Blatt M, Bastian P, Kagström B, Elmroth E, Dongarra J, Wasniewski J (2007). The iterative solver template library. Applied parallel computing—state of the art in scientific computing.

[CR7] Blatt M, Burchardt A, Dedner A, Engwer C, Fahlke J, Flemisch B, Gersbacher C, Gräser C, Gruber F, Grüninger C, Kempf D, Klöfkorn R, Malkmus T, Müthing S, Nolte M, Piatkowski M, Sander O (2016). The distributed and unified numerics environment, Version 2.4. Arch Numer Softw.

[CR8] Brazhnik PK, Tyson JJ (1999). On traveling wave solutions of fisher’s equation in two spatial dimensions. SIAM J Appl Math.

[CR9] Caragher S, Chalmers AJ, Gomez-Roman N (2019). Glioblastoma’s next top model: Novel culture systems for brain cancer radiotherapy research. Cancers.

[CR10] Chang EL, Akyurek S, Avalos T, Rebueno N, Spicer C, Garcia J, Famiglietti R, Allen PK, Chao KC, Mahajan A, Woo SY, Maor MH (2007). Evaluation of peritumoral edema in the delineation of radiotherapy clinical target volumes for glioblastoma. Int J Radiat Oncol Biol Phys.

[CR11] Conte M, Gerardo-Giorda L, Groppi M (2020). Glioma invasion and its interplay with nervous tissue and therapy: A multiscale model. J Theo Biol.

[CR12] Cook PA, Bai Y, Nedjati-Gilani S, Seunarine KK, Hall MG, Parker GJ, Alexander DC (2006) Camino: Open-source diffusion-mri reconstruction and processing. URL http://www.cs.ucl.ac.uk/research/medic/camino/files/camino_2006_abstract.pdf

[CR13] Corbin G, Hunt A, Klar A, Schneider F, Surulescu C (2018). Higher-order models for glioa invasion: from a two-scale description to effective equations for mass density and momentum. Math Models Meth Appl Sci.

[CR14] Engwer C, Hillen T, Knappitsch M, Surulescu C (2015). Glioma follow white matter tracts: a multiscale dti-based model. J Math Biol.

[CR15] Engwer C, Hunt A, Surulescu C (2016). Effective equations for anisotropic glioma spread with proliferation: a multiscale approach. J Math Med Biol.

[CR16] Fischer RA (1937). The wave of advance of advantageous genes. Ann Eug.

[CR17] Hunt A (2018) Dti-based multiscale models for glioma invasion. doctoralthesis, Technische Universität Kaiserslautern, URL http://nbn-resolving.de/urn:nbn:de:hbz:386-kluedo-53575

[CR18] Hunt A, Surulescu C (2017). A multiscale modeling approach to glioma invasion with therapy. Vietnam J Math.

[CR19] Jan Kelkel CS (2011). On some models for cancer cell migration throughtissue networks. Math Biosci Eng.

[CR20] Jbabdi S, Mandonnet E, Duffau H, Capelle L, Swanson KR, Pélégrini-Issac M, Guillevin R, Benali H (2005). Simulation of anisotropic growth of low-grade gliomas using diffusion tensor imaging. Magn Res Med.

[CR21] Kolmogoroff A, Petrovsky I, Piscounoff N (1988) Study of the diffusion equation with growth of the quantity of matter and its application to a biology problem. In: Pelcé P (ed) Dynamics of Curved Fronts, Academic Press, San Diego, pp 105 – 130 10.1016/B978-0-08-092523-3.50014-9

[CR22] Konukoglu E, Clatz O FAU Menze BH, Menze BH FAU Stieltjes B, Stieltjes B FAU Weber MA, Weber MA FAU Mandonnet E, Mandonnet E FAU Delingette H, Delingette H FAU Ayache N, N A (2010) Image guided personalization of reaction-diffusion type tumor growth models using modified anisotropic eikonal equations. IEEE Trans Med Imaging 10.1109/TMI.2009.2026413 19605320

[CR23] Konukoglu E, Sermesant M, Clatz O, Peyrat JM, Delingette H, Ayache N (2007) A recursive anisotropic fast marching approach to reaction diffusion equation: Application to tumor growth modeling. Information Proces Med Imag, 687–699 10.1007/978-3-540-73273-0_57 17633740

[CR24] Konukolu E, Clatz O, Bondiau PY, Delingette H, Ayache N, Larsen R, Nielsen M, Sporring J (2006). Extrapolating tumor invasion margins for physiologically determined radiotherapy regions. Medical Image Computing and Computer-Assisted Intervention—MICCAI 2006.

[CR25] Mandonnet E, Delattre JY, Tanguy ML, Swanson KR, Carpentier AF, Duffau H, Cornu P, Van Effenterre R, Alvord EC, Capelle L (2003). Continuous growth of mean tumor diameter in a subset of grade ii gliomas. Ann Neurol.

[CR26] Murray JD (2007). Mathematical biology: an introduction.

[CR27] Oraiopoulou ME, Tzamali E, Tzedakis G, Liapis E, Zacharakis G, Vakis A, Papamatheakis J, Sakkalis V (2018). Integrating in vitro experiments with in silico approaches for glioblastoma invasion: the role of cell-to-cell adhesion heterogeneity. Sci Rep.

[CR28] Painter K, Hillen T (2013). Mathematical modelling of glioma growth: the use of diffusion tensor imaging (dti) data to predict the anisotropic pathways of cancer invasion. J Theo Biol.

[CR29] Patel V, Hathout L (2017). Image-driven modeling of the proliferation and necrosis of glioblastoma multiforme. Theo Biol Med Modell.

[CR30] Porz N, Bauer S, Pica A, Schucht P, Beck J, Verma RK, Slotboom J, Reyes M, Wiest R (2014) Multi-modal glioblastoma segmentation: Man versus machine. PLoS ONE 9(5):e96873. 10.1371/journal.pone.0096873 10.1371/journal.pone.0096873 PMC4013039 24804720

[CR31] Rutter EM, Stepien TL, Anderies BJ, Plasencia JD, Woolf EC, Scheck AC, Turner GH, Liu Q, Frakes D, Kodibagkar V, Kuang Y, Preul MC, Kostelich EJ (2017). Mathematical analysis of glioma growth in a murine model. Scientific Rep.

[CR32] Sathornsumetee S, Reardon DA, Desjardins A, Quinn JA, Vredenburgh JJ, Rich JN (2007). Molecularly targeted therapy for malignant glioma. Cancer.

[CR33] Silbergeld DL, Chicoine MR (1997). Isolation and characterization of human malignant glioma cells from histologically normal brain. J Neuros.

[CR34] Stensjøen AL, Solheim O, Kvistad KA, Hå bergSalvesen Ø, Berntsen EM,  AK (2015). Growth dynamics of untreated glioblastomas in vivo. Neuro-Oncol.

[CR35] Swanson KR, Rostomily RC, Alvord EC (2008). A mathematical modelling tool for predicting survival of individual patients following resection of glioblastoma: a proof of principle. British J Cancer.

[CR36] Swanson KR, Rockne RC, Claridge J, Chaplain MA, Alvord EC, Anderson AR (2011). Quantifying the role of angiogenesis in malignant progression of gliomas: in silico modeling integrates imaging and histology. Cancer Res.

[CR37] Tracqui P, Cruywagen GC, Woodward DE, Bartoo GT, Murray JD, Alvord EC (1995). A mathematical model of glioma growth: the effect of chemotherapy on spatio-temporal growth. Cell Prolif.

[CR38] van der Vorst HA (1992). Bi-cgstab: a fast and smoothly converging variant of bi-cg for the solution of nonsymmetric linear systems. SIAM J Sci Stat Comput.

